# Health and Comfort at Wembley

**Published:** 1924-06

**Authors:** Theodore Ruete


### HEALTH AND COMFORT AT WEMBLEY.

BY THEODORE RUETE.

Many people who would wish to inspect for themselves the wonders of their heritage displayed at Wembley, doubtless have been restrained from venturing thither by thoughts of toiling in and out of long lines of buildings through crowds which render unavoidable sometimes minor accidents, faintness, or utter weariness. It may be said at once that at Wembley such fears are practically groundless, all that foresight can suggest having been done there to prevent such eventualities or to alleviate them when they do occur. Wembley is so extremely spacious that the crowds are not noticeably great, and there is an entire absence of wearisome monotony about the disposal and grouping of the wonderful halls of the various sections of the British Commonwealth of Nations. Each of these palaces stands apart, distinct in architecture and setting. Feebler folk, moreover, may hire any of the innumerable odd little " railodok " 'buses, or bath-chairs, to convey them to any desired point, and they will find chairs or seats in plenty everywhere. On seats often cunningly hidden amid lovely gardens, by plashing fountains or lakes, or grouped around the stands of the several excellent bands, they may quietly muse over all they have just left, and gather strength for their next adventure.

### Hospital and Nursing Arrangements.

Nothing more engenders hunger than sight-seeing in the open, and for this healthy hunger wise provision on an enormous scale has been made at Wembley. Excellent meals and light refreshments of every description, at prices to suit all purses, can be enjoyed in comfort, amid beautiful surroundings in all parts of Wembley's grounds. From those grounds, too, false modesty has at last been banished, never to return, let us hope, and instead of inadequate, poky, little public conveniences, hidden in out-of-the-way corners, large roomy buildings for both sexes stand boldly dotted everywhere. Careful thought for mortals' comfort indeed is evidenced all round. Practical help is never far away at Wembley, and a main ambulance station with two branches and ten first-aid cabinets, all connected by telephone, are placed in various parts of the grounds. Three medical men, one of whom is always on duty, are in charge of the main ambulance station, which is placed near the south-west entrance and contains six beds each for men and women, and two single private wards. Its nursing staff consists of a part-time matron and a sister-in-charge, assisted by a fully trained staff nurse, with an admirably equipped surgery for small operations and a dispensary, which enable it to deal with most cases brought in by Wembley's two motor-ambulances and a wheeled stretcher. Should any patients be too seriously ill to return home, or be the victims of accidents requiring operation or special treatment, they are sent on to Willesden General Hospital. At the smaller stations, placed respectively north and east in the grounds, V.A.D. nurses are employed under medical supervision, and the first-aid cabinets are in charge of capable Red Cross officials. All attendance is free, of course, but an unobtrusive collecting-box ever makes its challenge to gratitude.

### The Care of Children.

There are two further particulars in which provision has been made for the ease and comfort of visitors. Everywhere throughout the grounds stand uniformed guides to direct passers-by and look out for breaches of good behaviour, while boy scouts stand ever ready to act as messengers, to supply information, or to lead folk to any desired portion of the grounds or buildings. For the mother, moreover, who must either stay away or drag her youngsters round with her till they become cross or irritable, the exhibition's day-nursery provides a wonderful boon. This infants' palace, in a lovely grove of trees, on the east side of the grounds, near the Amusement Park, should be seen by every woman-visitor with the love of children in her heart. It will do her good to see the cool, clean, dainty place, and the kind, efficient nurses busy caring for an average of thirty-five children per day, at a charge of sixpence per child for four hours. On Saturdays the number of occupants has been mounting lately to as many as fifty, but there is accommodation for one hundred children within its kindly walls. No baby of less than three weeks is accepted (there has actually been an infant of that tender age there); nor can any girl over eight or any boy over six years be taken in charge. Light meals of biscuits and milk, and even an egg, if the mother so desires, are provided, for the centre has its tiny kitchen. The tiny guests are quite comfortable in the cots of an airy dormitory, or playing happily with toys or with other children in the roomy nursery, the big verandah, or the garden under the trees. A matron and two trained nurses and ten partly paid V.A.D. weekly volunteers have charge of this charming infants' castle, which in its completeness and fresh, innocent beauty is one of Wembley's most interesting features.

### The Wonders of Wembley.

To attempt to write of the many wonders of Wembley would be impossible here. The like of this amazing undertaking has never been seen, but those whose interests are of a domestic nature should pay special attention to the splendid models illustrating home life in Canada, Australia and New Zealand. Those of the former country are especially ingenious, the colourings and shapes of the various objects in the depicted scenes being entirely produced by the use of different seeds and grains. Many people will naturally also want to see the Queen's Dolls' House, in the Palace of Arts, on the western side, and the colossal figure of the Prince of Wales, in the yard of his Canadian Ranch, made solely of butter. The model dairy near the massive British Government building, too, where milking is done entirely by machinery, naturally will attract many, as will the processes for making buttons, hair-combs, electric light bulbs, etc., out of milk-casein, and the examples of electricity as applied to farming shown at the western end of Quality Street. Here, too, are many models, or parts, of dwelling-houses, all kinds of full-sized examples of which are scattered likewise all over the eastern end of the grounds. Nature lovers, moreover, will revel in the efforts of the landscape gardeners.

